# A novel polymorphism in the 1A promoter region of the vitamin D receptor is associated with altered susceptibilty and prognosis in malignant melanoma

**DOI:** 10.1038/sj.bjc.6602006

**Published:** 2004-07-06

**Authors:** J A Halsall, J E Osborne, L Potter, J H Pringle, P E Hutchinson

**Affiliations:** 1 Department of Cancer Studies and Molecular Medicine, University of Leicester, Leicester LE2 7LX, UK; 2 Department of Dermatology, Leicester Royal Infirmary, Leicester LE1 5WW, UK

**Keywords:** VDR, promoter polymorphism, malignant melanoma

## Abstract

The association of Taq 1 and Fok 1 restriction fragment length polymorphisms of the *vitamin D receptor* with occurrence and outcome of malignant melanoma (MM), as predicted by tumour (Breslow) thickness, has been reported previously. We now report a novel adenine–guanine substitution −1012 bp relative to the exon 1a transcription start site (A-1012G), found following screening by single-stranded conformational polymorphism of this promoter region. There was a total of 191 MM cases , which were stratified according to conventional Breslow thickness groups, cases being randomly selected from each group to form a distribution corresponding to the known distribution of Breslow thickness in our area, and this population (*n*=176) was compared to 80 controls. The A allele was over-represented in MM patients and, with GG as reference, odds ratio (OR) for AG was 2.5, 95% confidence interval (CI) 1.1–5.7, (*P*=0.03) and AA 3.3, CI 1.4–8.1, (*P*=0.007). The outcome was known in 171 of 191 patients and the A allele was related to the development of metastasis, the Kaplan–Meier estimates of the probability of metastasis at 5 years being: GG 0%; AG 9%, CI 4–16%; AA 21%, CI 12–36%; (*P*=0.008), and to thicker Breslow thickness groups (*P*=0.04). The effect on metastasis was independent of tumour thickness and A-1012G may have predictive potential, additional to Breslow thickness. Neither the Fok 1 nor Taq 1 variants (f and t) were significantly related to the development of metastasis, although there was a strong relationship of fftt with the thickest Breslow thickness group (*P*=0.005). There was an interaction between the A-1012G and Fok 1 polymorphisms (*P*=0.025) and the Fok 1 variant enhanced the effect of the A allele of the A-1012G polymorphism on metastasis, the probability of metastasis for AAff at 5 years follow-up being 57%, CI 24–92%.

The polymorphisms of the *vitamin D receptor (VDR)*, reported in the literature, comprise a cluster of tightly linked polymorphisms at the 3′-end and two polymorphisms at the 5′-end of the gene. The 3′ polymorphisms are Apa 1 (Faraco *et al*, 1989) and Bsm 1 (Morrison *et al*, 1992) in intron 8, Taq 1(Morrison *et al*, 1992) in a silent site in exon 9 and a length polymorphism of a polyadenyl (polyA) microsatellite in the 3′-untranslated region (Ingles *et al*, 1997b), classified into long (L) and short (S) variants (L demonstrates linkage disequilibrium with b, a, T). The 5′ polymorphisms are Fok 1(Saijo *et al*, 1991) situated in exon 2, 10 base pairs upstream from an ATG translation start point, and a recently described polymorphism in the promoter region, situated at −3731 bp relative to the exon 1a transcription start site (Arai *et al*, 2001) within a binding element of Cdx-2, which is a caudal-related homeodomain transcription factor. The 3′ region polymorphisms do not affect VDR protein structure, while Fok 1 (C–T transition) alters an ACG codon resulting in a further upstream start codon and a three amino-acid extended protein (Saijo *et al*, 1991). Both the 3′ and Fok 1 polymorphisms have been reported to be functional in terms of VDR transactivation (Whitfield *et al*, 2000). Cdx-2 is important during the development of the intestine and in adults it has been shown to regulate VDR expression in the small intestine (Yamamoto *et al*, 1999). The expression of Cdx-2 has also been found in other tissues such as the brain and prostate. Polymorphism at the Cdx-2-binding site significantly alters the transcriptional activity of the *VDR* promoter region (Arai *et al*, 2001).

Since 1996, there have been many reports of associations of polymorphisms of the *VDR* with systemic carcinomas. The 3′ polymorphisms have been reported to be associated with the occurrence and outcome, as assessed by metastasis or presence of adverse prognostic markers, of prostatic cancer (Taylor *et al*, 1996; Ingles *et al*, 1997c; Ingles *et al*, 1998; Ma *et al*, 1998; Habuchi *et al*, 2000; Hamasaki *et al*, 2001; Medeiros *et al*, 2002), breast cancer (Curran *et al*, 1999; Lundin *et al*, 1999; Ingles *et al*, 2000; Bretherton-Watt *et al*, 2001; Cui *et al*, 2001; Schondorf *et al*, 2003) and renal cancer (Ikuyama *et al*, 2002). Fok 1 polymorphisms have been reported to be associated with the outcome of prostate cancer (Hamasaki *et al*, 2002) and occurrence of breast (Ingles *et al*, 1997a) and colon (Wong *et al*, 2003) cancers. However, other studies have failed to find an association with prostate cancer (Jenkins *et al*, 1997; Dunning *et al*, 1999; Luscombe *et al*, 2001) or breast cancer (Correa-Cerro *et al*, 1999; Furuya *et al*, 1999; Watanabe *et al*, 1999; Blazer *et al*, 2000; Chokkalingam *et al*, 2001), but many of these (Jenkins *et al*, 1997; Correa-Cerro *et al*, 1999; Furuya *et al*, 1999; Watanabe *et al*, 1999; Blazer *et al*, 2000) were relatively limited studies of approximately 100 or less cases. The Cdx-2 polymorphism has been linked with increased risk in cancer of the prostate (Bodiwala *et al*, 2004).

We have previously reported an association with the occurrence and, particularly, the outcome of malignant melanoma (MM), as assessed by Breslow tumour thickness and polymorphisms at the Fok 1 and Taq 1 restriction sites (ttff) (Hutchinson *et al*, 2000). We now describe a new polymorphism in the promoter region upstream of the exon 1a transcription start site, A-1012G. We report investigations into the relationship of the A-1012G and A-1012G/Taq 1 and A-1012G/Fok 1 genotype combinations with the occurrence and outcome of MM, in terms of the development of metastasis and as predicted by Breslow thickness and compare the strength of the A-1012G polymorphism with the corresponding relationships of Taq 1 and Fok 1.

## MATERIALS AND METHODS

### Single-stranded conformational polymorphism (SSCP) analysis

All oligonucleotide primers used for PCR are shown in Table 1

**Table 1:** Oligonucleotide sequences used as PCR primers

**Primer pair**	**Forward primer**	**Reverse primer**	**Amplicon position (bp)**
1A/Dp1	CTGATGACGGCATGTGCT	CAGCCTTTGTTGGAGGAGAG	−2119 to −1755
1A/Dp2	CAGTGGGATGTGCAGAGAGA	GCTAGCGGTGATTCTTGTGG	−1979 to −1529
1A/Dp3	AGATGTCAGGCCAGTCAAGC	GGTATCATGGCAACTTTCTGG	−1676 to −1329
1A/Dp4	ATGGTCCATTCCCAAGTTCA	CAGAGGGACAAGGTGAAAGG	−1411 to −1051
1A/Dp5	AGCAGATTTGCTGGGCTCTA	TGCTTCCCTTGACTGTGTGA	−1163 to −818
1A/Dp6	TCCCACTGCACAGTGAGTTC	AAGTGGAAACCGGAGTTGC	−914 to −554
1A/Dp7	GATATCGGGTGGGAGCAAT	TGGGACAGAGTTGTCGATGA	−594 to −232
1A/Dp8	ACAGGCTGAAGCGGGTATC	CCGAGTCCCTATCCTGAGAC	−281 to +126
1A/Dp9	GCAAGAGAGGACTGGACCTG	GCGGAGCATTTCTCCTAAGC	−94 to +334
1A/Dp10	TCTCAGCGGTAAACTTGGCTA	AGACCCAACTCCACCATCAC	+186 to +535
SNP1	CCTCCTCTGTAAGAGGCGAATAGCGAT	GGACAGGTGAAAAAGATGGGGTTC	−1039 to −861
Fok 1	CTGGCACTGACTCTGGCTCT	TGCTTCTTCTCCCTCCCTTT	−48 to +199^a^
Taq 1	CAGAGCATGGACAGGGAGCAAG	CGGCAGCGGATGTACGTCTGCAG	−260 to +338^b^

PCR=polymerase chain reaction; bp=base pair. All amplicon positions are relative to the exon 1a start site except

a relative to the start of exon 2 and

b relative to the start of exon 9.

. A ∼2500 bp region from 120 bp upstream of exon 1e to 70 bp downstream of exon 1d including exon 1a was amplified by PCR in 10 overlapping amplicons of ∼350 bp (primers 1A/Dp1-10) for 35 cycles with an annealing temperature of 60°C in PCR buffer (45 mM Tris-HCl (pH 8.8), 11 mM (NH_4_)_2_SO_4_, 4.5 mM MgCl_2_, 110 *μ*g ml^−1^ BSA, 6.7 mM *β*-mercaptoethanol 4.4 *μ*M EDTA (pH 8.0), 200 *μ*M dNTPs). Each amplicon was screened for variation by SSCP in 36 control samples. A measure of 3 *μ*l PCR product was added to 9 *μ*l denaturing loading buffer (95% formamide, 0.25% bromophenol blue, 0.25% xylene cyanol, 10 mM sodium hydroxide) and heated to 95°C for 3 min, chilled on ice and loaded onto a 0.6–0.8 × MDE (BioWhittaker, 50620) gel, depending on amplicon size, in 0.6% TBE buffer and run at 500 V for 30 min followed by 270–350 V overnight, depending on amplicon size. Where variation was found, variant bands were reamplified from the gel and sequenced by Big Dye sequencing on an ABI prism 377 sequencer.

The possible effects of sequence variation on promoter activity were determined by the analysis of potential transcription factor-binding sites within variable regions using the TESS database (Schug and Overton, 1997).

### Genotype screening

To screen for the promoter polymorphism in patients and controls, a 150 bp region around the polymorphism was amplified using primer pair SNP1 (Table 1). PCR cycling was carried out with a 55°C annealing temperature for three cycles followed by 65°C for 37 cycles. The forward primer was adjacent to the polymorphism and mutated the sequence to introduce an *Eco*RV restriction site in the A allele but not in the G allele such that in subsequent *Eco*RV digestion (37°C, 20 h), followed by agarose-gel electrophoresis, the A allele was restricted and the G allele remained uncut. Fok 1 and Taq 1 were amplified with the appropriate primers (Hutchinson *et al*, 2000) for 35 cycles with an annealing temperature of 60°C. PCR products were then digested with Fok 1 (37°C, 20 h) or Taq 1 (65°C, 20 h). The Fok 1 F and Taq 1 T alleles were refractory to digestion, while the f and t alleles were restricted.

### MM studies

Patients with a diagnosis of MM who attended the Pigmented Lesion Clinic between 1995 and 1997 were recruited. Patients with lentigo maligna melanoma were not included. It was attempted to recruit all patients, but this was not always possible in busy clinics. Documented data were age at presentation, gender, skin type based on the Fitzpatrick classification (Fitzpatrick, 1988), eye colour, hair colour at age 21 years, tumour site and Breslow group and presence of metastases on follow-up. Breslow thickness (defined as the vertical thickness of the tumour from the granular layer of the epidermis to the deepest part of the melanoma) was determined by specialist pathologists. On the basis of Breslow thickness, patients were divided into five conventional Breslow thickness groups (0–4); *in situ*, <0.75 mm, 0.75–1.49 mm, 1.5–3.49 mm and ⩾3.5 mm (Mackie *et al*, 1985). For the occurrence studies, a subsample of patients were stratified according to Breslow thickness group to conform with frequencies within groups according to the reported distribution in the Leicestershire area (*n*=738) (Osborne and Hutchinson, 2001). Controls consisted of UK Caucasian patients not known to have cancer. This study was approved by the local Ethics Committee and written informed consent was given by the patients.

### Statistics

Occurrence and Breslow thickness data were analysed by contingency tables (Unistat Statistical Package, version 5.0, Unistat, UK) and logistic regression (Stata software package, version 7.0, Stata Corporation, Texas, USA). Metastatic data were analysed using the Kaplan–Meier product limit estimator (Unistat), with the log-rank comparison statistic, and by the Cox's proportional-hazard model (Stata). Interactions were assessed by the Cox's likelihood-ratio test (Stata).

## RESULTS

### Promoter screening

**Figure 1:**
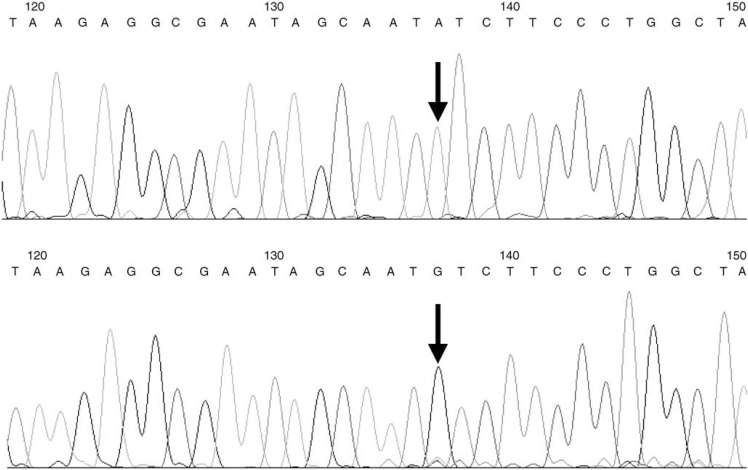
Sequencing traces of the region around the polymorphism, the variable base is marked by an arrow.

Single-stranded conformational polymorphism revealed variation in the 1A/Dp5 region (−1163 to −818 bp). Sequencing of the variant bands revealed an A–G polymorphism, at −1012 bp relative to the exon 1a transcription start site (Figure 1

). Therefore, the sequence in the region was either: −1020 ATAGC*AAT****A****TC*TTC or −1020 ATAGCAAT**G**TCTTC.

Analysis with the TESS database revealed a strong GATA-3 core-binding site at this locus in the A allele (given in italics above), which was not present on analysis of the G allele.

### MM studies

The percentages of MM in each Breslow thickness group presenting in Leicestershire in the past 10 years are as follows: *in situ*, 13.8%; <0.75 mm, 26.6%; 0.75–1.49 mm, 21.5%; 1.5–3.49 mm, 21.0% and ⩾3.5 mm, 17.1%. A total of 191 patients were recruited. The numbers of patients randomly chosen for allocation to the respective thickness groups from the 191 patients were 24 (13.6%), 49 (27.8%), 35 (19.9%), 38 (21.6%) and 30 (17.0%). The total number of MM patients for the occurrence study was therefore 176 (mean age 54.3 years, 110 females) and there were 80 controls (mean age 56.2 years, 40 females). Age or sex did not have a statistically significant relationship with *VDR* promoter genotype in either controls or MM patients. In the metastatic outcome part of the study, there was a total of 171 of 191 patients in whom the outcome was known.

Table 2

**Table 2:** Allele and genotype frequencies of A-1012G in MM patients and controls

	**Alleles**		**Genotypes**		
	**A**	**G**	**AA**	**AG**	**GG**
Controls	86 (54%)	74 (46%)	22 (27%)	42 (52%)	16 (20%)
MM patients	244 (64%)	140 (36%)	66 (38%)	92 (53%)	16 (9%)
	*P*=0.03		*P*=0.03		

MM=malignant melanoma.

shows allele and genotype frequencies in controls and MM patients. Genotype frequencies conformed to the Hardy–Weinberg equilibrium in both subject groups. The A allele was over-represented in the MM patients (*P*=0.03). Similarly, AA genotype was more and GG less frequent in MM patients (*P*=0.03). Correcting for age and sex and with GG as reference, odds ratio (OR) for AG was 2.5, 95% confidence interval (CI) 1.1–5.7 (*P*=0.03) and AA 3.3, CI 1.4–8.1, (*P*=0.007).

**Figure 2:**
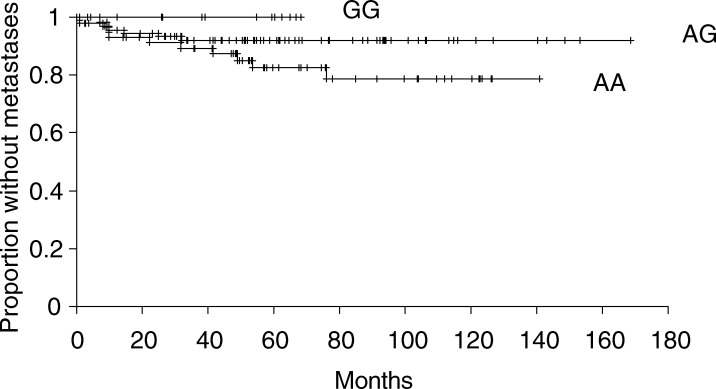
Kaplan–Meier estimates of the probability of metastasis for A-1012G genotypes.

The mean time to metastasis was 31 months (range 1–171 months) (*n*=19) and mean follow-up time in patients not developing metastasis was 75 months (3–255 months) (*n*=152). Figure 2

shows Kaplan–Meier curves for metastasis-free times for the promoter genotypes, which differed significantly (*P*=0.008). The Kaplan–Meier estimates of the probability of metastasis at 5 years follow-up were: GG 0%; AG 9%, CI 4–16%; AA 21%, CI 12–36%. The results of Cox's proportional-hazard regression analysis are shown in Table 3

**Table 3:** Results of Cox's proportional-hazard regression analysis (univariate) of metastatic rate on A-1012G, Fok 1 and Taq 1 genotypes, and on A-1012G genotype combinations with Fok 1 and Taq 1

**Polymorphisms**	**Genotype**	**Number**	**HR**	**95% CI**	**Significance**
A-1012G	GG/AG	110	1		
	AA	59	**2.9**	1.1–7.5	*P*=0.03
					
Fok	FF/Ff	140	1		
	Ff	30	1.3	0.4–4.1	*P*=0.6
					
Taq	TT/Tt	143	1		
	Tt	26	1.5	0.5–4.6	*P*=0.5
					
Fok/Taq	Other genotypes	165	1		
	ff/tt	4	2.2	0.3–16.3	*P*=0.5
					
A-1012G/Taq	Other genotypes	130	1		
	AATT	27	2.5	0.9–7.4	*P*=0.1
	Aatt	11	3.1	0.8–11.2	*P*=0.1
					
A-1012G/Fok	Other genotypes	130	1		
	AAFF	30	**4.3**	1.5–12.4	*P=*0.006
	AAff	9	**8.6**	2.5–29.6	*P=*0.001
	AAFF/Ff	50	1		
	AAff	9	**4.7**	1.2–18.6	*P*=0.03

HR=hazard ratio; CI=95% confidence interval for hazard ratio estimate. Bold numerals represent statistically significant results.

. As there were no metastases with genotype GG, hazard ratios (HR) for AA and AG compared with GG were not reliable and therefore AA *vs* AG/GG are shown, confirming a significantly worse prognosis for AA. Inclusion of the covariates, age at onset, male gender, skin type, eye and hair colour and lesional site made no significant impact on this (results not shown).

Conversely, the A-1012G polymorphism was not strongly associated with Breslow thickness (*n*=191), although for Breslow thickness groups two, three and four combined *vs* groups zero and one combined (⩾1.5 mm *vs* <1.5 mm depth); OR for AA *vs* other genotypes was 1.9 (CI 1.0–3.6, *P*=0.04). The relationship of promoter polymorphism and metastasis was reinvestigated correcting for Breslow thickness, when HR for AA *vs* any other genotype was 2.7, which was very similar to the uncorrected value of 2.9, suggesting that the effect of the polymorphism on metastasis was largely independent of depth of invasion. Table 4

**Table 4:** Proportion of patients developing metastases in relation to Breslow group of the melanoma and A-1012G genotype

	**Breslow thickness group**				
	***In situ***	**<0.75 mm**	**0.75**–**1.49 mm**	**1.5**–**3.49 mm**	**⩾3.5 mm**
GG	0% (0/3)	0% (0/5)	0% (0/3)	0% (0/4)	0% (0/2)
AG	0% (0/13)	0% (0/30)	0% (0/17)	17% (3/18)	27% (4/15)
AA	0% (0/7)	8% (1/13)	12% (2/16)	14% (2/14)	67% (6/9)

()=number of patients developing metastasis/total number patients in each group.

shows the proportion of patients in each promoter genotype/Breslow thickness group who developed metastasis. The highest proportion occurred in patients who had both AA genotype and the thickest tumours (Breslow thickness ⩾3.5 mm, group 4). However, some patients with thin tumours developed metastasis but only in the presence of the AA genotype.

The Taq 1 homozygote variant (tt) was associated with Breslow thickness groups three and four combined (Breslow thickness ⩾1.5 mm) when corrected for age at presentation, sex, skin type eye and hair colour and site of MM (OR 3.1 CI 1.2–8.3, *P*=0.02) but the Fok 1 homozygote variant (ff) was not (OR 1.4, CI 0.6–3.5, *P*=0.5). The fftt genotype combination was strongly associated with the thickest Breslow thickness group, group 4 (OR 24, CI 3–225, *P*=0.005). Similarly, tt, ff and ttff were not significantly associated with poorer prognosis in terms of metastasis (Table 3).

**Figure 3:**
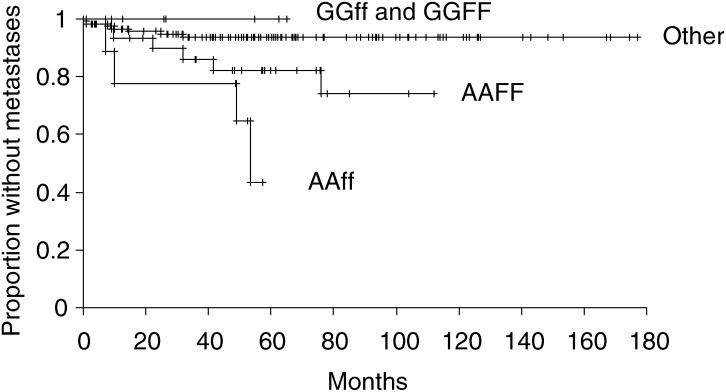
Kaplan–Meier estimates of the probability of metastasis for A-1012G/Fok 1 combination genotypes.

There was some correlation between A-1012G with Fok 1 polymorphisms (F and A), Spearman's rank correlation 0.15 (*P*=0.01) but not with Taq 1 (*P*=0.5). There was a statistically significant interaction of the A-1012G with Fok 1 polymorphisms (*P*=0.025) but not with Taq 1. Considering A-1012G/Fok 1 and A-1012G/Taq 1 genotype combinations (Figure 3

), the Kaplan–Meier estimates of the probability of metastasis at 5 years follow-up were AAff 57%, CI 24–92%; AAFF 18%, CI 8–38%; GGff 0%; GGFF 0% and all other genotypes 6%, CI 3–13%, (*P*=0.001). On Cox's proportional-hazard model regression (Table 3), there was a strong effect of AAff, which was associated with a greater metastatic rate than AAFF/Ff (HR 4.7, *P*=0.03) when corrected for the other covariates. This difference was greater than the corresponding comparison of ff *vs* FF/Ff (HR 1.3, *P*=0.6).

## DISCUSSION

This newly described polymorphism of the 1a promoter, A-1012G, has been found to be related to occurrence of MM (Table 2), the A allele being over-represented in the MM population. When investigating the effect of a variable on occurrence of a malignancy, it is necessary to employ a sample of patients which is representative in terms of severity of the disease in general if, as in this case, the variable is also related to the outcome. Failure to do this may be a cause of some of the conflicting results in the literature. The cases were therefore stratified according to Breslow thickness, which is the best single predictor of MM outcome.

The promoter polymorphism was clearly related to outcome as assessed by the development of metastasis (Figure 2, Table 3). There was a weaker relationship with thicker Breslow thickness groups. We have previously reported a relationship of Taq 1 and Fok 1 variant gene combination (ttff) with increased Breslow thickness (Hutchinson *et al*, 2000). This was true in the present study (*P*<0.005), but the ttff genotype combination was not significantly associated with metastasis.

The relationship between the A-1012G genotype and development of metastasis was also analysed by Cox's proportional-hazard regression, correcting for Breslow group, when the promoter genotype retained significance. The *VDR* would therefore appear to be related to both Breslow thickness and development of metastasis, but the effect on metastasis is at least partially independent of tumour thickness. This finding has potential importance in the interpretation of molecular mechanism of tumour spread. Also, the inclusion of the *VDR* promoter genotype when predicting MM outcome with Breslow thickness group should potentially enhance precision (Table 4). The highest proportion of metastases occurred in patients who had both AA genotype and the thickest tumours (Breslow thickness group 4). However, some thinner tumours developed metastasis, but usually in the context of AA genotype. Studies on a larger scale are required to further investigate the predictive capacity of this polymorphism.

Neither the Fok 1 nor Taq 1 polymorphism was significantly related to the development of metastasis. However, the promoter and Fok 1 combination genotype (AAff) was greatly associated with a higher propensity to metastasis (Table 3). The Fok 1 polymorphism appears to augment the effect of the promoter polymorphism, as evidenced by a significant statistical interaction on Cox's regression and a significant difference in prognosis associated with AAff compared with AAFf/AAFF genotype combinations (Table 3), despite no similar difference between ff and Ff/FF.

The mechanism of action of this promoter polymorphism is not known. One possibility is that the polymorphism modulates docking of a transcription factor. The analysis of this region with the TESS database (Schug and Overton, 1997) demonstrates that the polymorphism is within the core sequence of a likely GATA-3-binding site in the A allele, while this binding site is not present in the G allele. Merika and Orkin, (1993) demonstrated that DNA strands containing the core sequence AGATAT (the reverse orientation of the A allele) bound human GATA-3, while zero of 63 sequences shown to bind GATA-3 contained the core sequence AGACAT (the reverse orientation of the G allele). GATA-3 is an important transcription factor directing the polarisation of naïve T cells to Th-2 cells (Rengarajan *et al*, 2000). 1*α*,25-dihydroxyvitamin D_3_ has been shown to upregulate GATA-3 gene expression and the GATA-3 protein promotes polarisation to Th-2 (Boonstra *et al*, 2001). The present findings would suggest that the GATA-3 response element in the A allele of the *VDR* promoter may produce a positive feedback loop and amplify the GATA-3-induced polarisation. Therefore, this polymorphism may influence immune response to cancer, particularly in cancers, such as MM, which show high expression of MAGE antigens. Such tumours are susceptible to Th-1 responses (Tatsumi *et al*, 2002). The argument would be that the A allele is associated with a Th-1 to Th-2 switch, which reduces the Th-1 cytotoxic response to cancer cells. A further possibility is that A-1012G is a determinant of whether transcription is initiated in exon 1a or 1d. Exon 1d contains an alternative start codon, which can lead to the expression of a significantly N-terminally extended protein (VDRB1). This is reported to have greater transactivation potential than the short protein, translated at the conventional start codon in exon 2 (VDRA) (Gardiner and Eisman, 2003), although this has not been consistently reported (Sunn *et al*, 2001). The majority of evidence is that vitamin D and the VDR have a protective effect in cancer (Osborne and Hutchinson, 2002). Therefore, if A-1012G were determining the transcription start site, then the G allele would be expected to be associated with the VDRB1 protein and the A allele with the shorter VDRA protein, which would be further altered by the f allele of the Fok 1 polymorphism. This would be compatible with the finding of enhanced effect of AAff over AA found in the present study.

In conclusion, the novel *VDR* promoter polymorphism, A-1012G, is related to MM occurrence and outcome as predicted by Breslow thickness but more particularly with the development of metastasis. This relationship is considerably stronger than the relationships of the Taq 1 and Fok 1 polymorphisms and their genotype combinations. The Fok 1 variant enhanced the effect of the A-1012G/Fok 1 polymorphism on metastasis. The effect on metastasis is at least partially independent of tumour thickness and A-1012G may have predictive potential in addition to Breslow thickness.
